# Association of preoperative albumin-corrected anion gap with 28-day mortality in cardiac surgery patients: a retrospective cohort study

**DOI:** 10.1186/s12872-026-05779-9

**Published:** 2026-03-27

**Authors:** Haiyang Hu, Zhibo Zhang, Wangping Peng, Pengfei Du, Wenkui Yu

**Affiliations:** 1 Department of Critical Care Medicine, Nanjing Drum Tower Hospital, Affiliated Hospital of Medical School, Nanjing University, Nanjing, China; 2 Department of Critical Care Medicine, Nanjing Drum Tower Hospital, Drum Tower Clinical College, Nanjing University of Chinese Medicine, Nanjing, China; 3 Department of Critical Care Medicine, Affiliated Hospital of Jiangnan University, Wuxi, China

**Keywords:** Cardiac surgery, 28-day mortality, Albumin-corrected anion gap, MIMIC-IV, Prognosis, Risk stratification

## Abstract

**Background:**

Cardiac surgery patients face significant postoperative risks. The albumin-corrected anion gap (ACAG) has emerged as a potential prognostic marker, but its role in predicting outcomes following cardiac surgery remains understudied. This study examined the association between preoperative ACAG levels and 28-day mortality following ICU admission in patients undergoing cardiac surgery.

**Methods:**

This retrospective cohort study analyzed 5,006 cardiac surgery patients from the MIMIC-IV database. Patients were categorized based on ACAG levels: low (< 12 mmol/L), normal (12–20 mmol/L), and high (> 20 mmol/L). Hierarchical Cox proportional hazards models, restricted cubic spline (RCS) analysis, and exploratory mediation analysis were used to evaluate the association between preoperative ACAG and 28-day mortality following ICU admission. Incremental predictive analyses were additionally performed to assess whether ACAG improved the prognostic performance of models based on SOFA and SAPS II.

**Results:**

Elevated ACAG levels (> 20 mmol/L) were significantly associated with a heightened risk of 28-day mortality following ICU admission across all models. In the most comprehensively adjusted model, the hazard ratio was 1.049 (95% CI 1.024–1.075, *P* < 0.001). ACAG demonstrated superior predictive value for 28-day mortality following ICU admission compared to the traditional anion gap (AUC 0.776 vs. 0.690). RCS revealed a nonlinear relationship, with mortality risk sharply increasing at ACAG levels above 20 mmol/L.In incremental predictive analyses, adding ACAG improved the prognostic performance of both SOFA- and SAPS II-based models. Exploratory mediation analyses suggested that SAPS II, GCS, and several electrolyte-related variables accounted for part of the observed association between preoperative ACAG and 28-day mortality.

**Conclusions:**

Elevated preoperative ACAG levels were associated with increased 28-day mortality risk in cardiac surgery patients. Although part of this association may reflect overall illness severity and postoperative physiological stress, ACAG may serve as a useful early risk-stratification marker and could complement existing prognostic assessment frameworks. However, its causal role cannot be inferred from this observational study, and prospective studies are needed to validate these findings.

**Supplementary Information:**

The online version contains supplementary material available at 10.1186/s12872-026-05779-9.

## Introduction

Cardiac surgery remains a cornerstone in the treatment of various cardiovascular diseases, offering life-saving interventions for millions of patients worldwide [1]. Despite significant advancements in surgical techniques and perioperative care, postoperative outcomes continue to pose substantial risks, including mortality and morbidity [2]. The persistence of adverse clinical outcomes, particularly in patients with metabolic disturbances, underscores the need for more refined prognostic tools and targeted interventions [3].

Recently, the role of acid-base balance in forecasting postoperative outcomes has attracted increasing attention. The anion gap (AG), a key indicator of acid-base equilibrium, has traditionally been used to assess metabolic acidosis [4]. Furthermore, it is considered a crucial factor influencing postoperative outcomes in cardiac surgery patients [5]. However, AG levels are susceptible to fluctuations in serum albumin concentrations [4]. Consequently, the albumin-corrected anion gap (ACAG) has emerged as a more precise measure of internal acid-base status, garnering attention for its potential associations with various adverse outcomes [6, 7].

Although the clinical importance of ACAG has been examined across different medical conditions, its precise function in patients undergoing cardiac surgery is still underexplored [8]. The recovery outcomes following heart surgery are shaped by numerous elements, such as metabolic disorders and disruptions in acid-base balance [9, 10]. Therefore, a thorough investigation into the predictive value of ACAG in this particular population is crucial.

This study aimed to address this knowledge gap by investigating the association between preoperative ACAG and 28-day mortality following ICU admission in patients undergoing cardiac surgery. Using a large critical care database, we further evaluated whether ACAG provides incremental prognostic information beyond established severity scores and may serve as an early risk-stratification marker for identifying high-risk individuals. These findings may offer additional insight into the potential role of ACAG in preoperative risk assessment and prognostic evaluation in cardiac surgery patients.

## Methods

### Data source

In this retrospective cohort analysis, we employed data obtained from the Medical Information Mart for Intensive Care IV (MIMIC-IV, v3.0) database. This publicly accessible, deidentified dataset is provided by the Beth Israel Deaconess Medical Center (BIDMC) located in Boston, MA [11, 12]. MIMIC-IV contains data from over 364,627 unique patients, with 94,458 ICU stays and 546,028 hospital admissions occurring between 2008 and 2022. The dataset integrates information from both the hospital-wide electronic health record system and ICU-specific clinical information systems, organized in a modular format that facilitates easy linkage with external data sources. The data includes patient demographics, laboratory measurements, medications, and detailed clinical notes, making it a robust resource for conducting comprehensive healthcare research. One of the study authors [Haiyang Hu] obtained full access to the MIMIC-IV database and extracted the necessary data for this study under certification number [Certification Number:64961191]. The use of MIMIC-IV was approved by the Institutional Review Board (IRB) at BIDMC, with a waiver for individual patient consent, as the data is fully deidentified in accordance with HIPAA regulations. This study is reported in accordance with the STROBE guidelines for observational research [13].

### Selection of participants

**Fig. 1:**
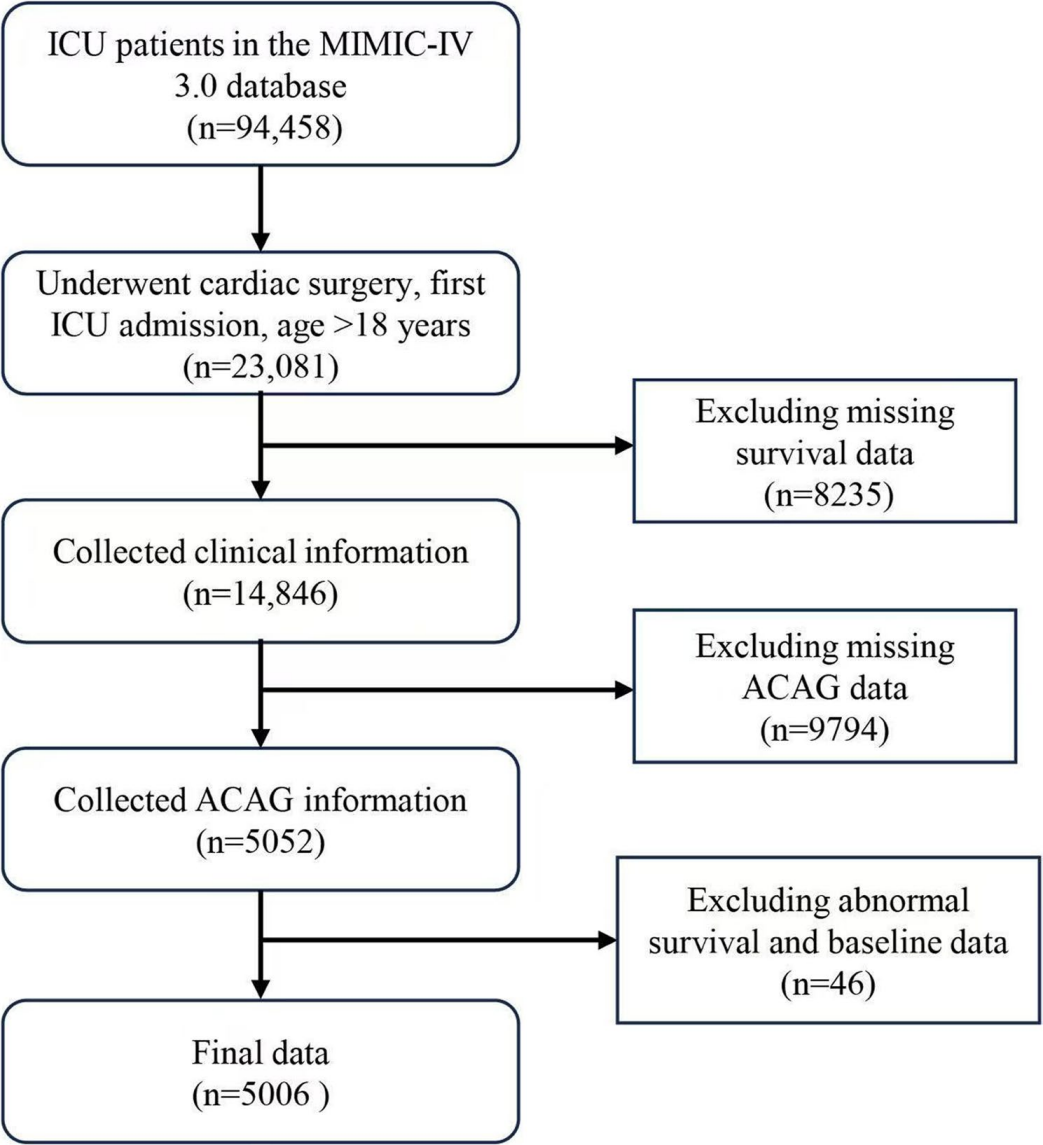
Flowchart of the study population selection process. The diagram outlines the steps from initial identification of ICU patients in the MIMIC-IV database to the final cohort of 5,006 patients included in the analysis after applying exclusion criteria

The inclusion criteria for this study were as follows (Fig. 1):

1. Patients who underwent cardiac surgery, identified by International Classification of Diseases (ICD) codes (see Supplemental Table S1).
2. Patients aged 18 years or older.
3. Patients admitted to the ICU for the first time.

The exclusion criteria were as follows:

1. Patients with missing survival data.
2. Patients with missing ACAG information.
3. Patients with abnormal survival or baseline data due to medical record errors.

Data extraction was conducted using Structured Query Language (SQL), considering only the first ICU admission for patients with multiple admissions to ensure data accuracy and consistency.

### Variable extraction

The primary exposure in this study was ACAG, calculated as AG (mmol/L) + [4.4 − albumin (g/dL)] × 2.5. Preoperative ACAG was calculated using the initial preoperative anion gap and serum albumin measurements obtained upon hospital admission, prior to surgical intervention, and extracted from the MIMIC-IV hospital laboratory records (hosp.labevents linked to d_labitems). Patients without the required preoperative anion gap or albumin measurements for ACAG calculation were excluded before cohort entry. Based on ACAG levels, participants were categorized into three groups: normal ACAG (12–20 mmol/L), high ACAG (> 20 mmol/L), and low ACAG (< 12 mmol/L). The primary outcome was 28-day all-cause mortality following ICU admission. Survival time was calculated from ICU admission to death or censoring within 28 days. To accurately reflect the patients’ physiological status at different clinical stages, distinct time windows were applied for data extraction. The primary exposure variable, Preoperative ACAG, was calculated using the initial laboratory measurements obtained upon hospital admission (prior to surgical intervention). In contrast, clinical covariates, including vital signs and critical illness severity scores (e.g., SOFA and SAPS II), were extracted from the data recorded during the first 24–48 h following ICU admission (postoperative phase). This design allows us to examine the association between preoperative baseline metabolic status and 28-day mortality, while additionally testing whether this association remains robust after accounting for postoperative disease severity in extended models.

In addition to demographic data (age, body mass index, sex, race, marital status, insurance status) and comorbidities (chronic kidney disease (CKD), heart failure (HF), myocardial infarction (MI), ischemic heart disease (IHD), hypertension (HTN), stroke, chronic obstructive pulmonary disease (COPD), type 1 diabetes (T1DM), type 2 diabetes (T2DM), cancer), key covariates assessed within 48 h of ICU admission were included. Based on ICD procedure codes, cardiac surgeries were classified into four categories: arrhythmia device implantation, coronary artery surgery, other cardiac surgery, and valve surgery. These covariates encompassed laboratory results (e.g., blood urea nitrogen, creatinine, hemoglobin, red blood cell count, platelet count, sodium, chloride, glucose, potassium, neutrophils, lymphocytes, albumin, total protein, C-reactive protein and white blood cell count), clinical scores (e.g., Glasgow Coma Scale (GCS), Sequential Organ Failure Assessment (SOFA), Simplified Acute Physiology Score II (SAPSII)), vital signs (e.g., heart rate, respiratory rate, oxygen saturation, body temperature (Fahrenheit), non-invasive blood pressure monitoring), and treatment measures (e.g., mechanical ventilation, use of NSAIDs, antihypertensive medications, antibiotics, corticosteroids). Additionally, the incidence of acute kidney injury (AKI) within seven days of ICU admission was evaluated. Variables with more than 20% missingness were excluded from the multivariable analyses. Missing covariate data were handled using multiple imputation by chained equations with the mice package. Continuous variables were imputed using predictive mean matching. Importantly, the primary exposure variable (preoperative ACAG) was not imputed; instead, patients lacking the required preoperative albumin or anion gap measurements were excluded prior to analysis. Estimates from the imputed datasets were pooled using Rubin’s rules.

### Statistical analyses

Normality tests were first conducted on continuous variables. D Normally distributed variables are presented as mean ± standard deviation and compared using the t-test. Non-normally distributed variables are shown as median (IQR) and compared using the Mann-Whitney U test. Categorical variables are expressed as percentages and compared with the χ² test.

To assess the 28-day survival differences among the three groups, Kaplan–Meier (KM) curves were first applied. Receiver operating characteristic (ROC) curves were then constructed to compare the predictive ability of ACAG and AG as continuous variables for 28-day outcomes, with the area under the curve (AUC) used as the comparison metric. To further assess whether preoperative ACAG provided prognostic information beyond established ICU severity scores, we constructed baseline models using SOFA and SAPS II separately and compared them with corresponding extended models that additionally incorporated preoperative ACAG. Model discrimination was evaluated using the AUC, and differences between paired AUCs were compared using the DeLong test. In addition, continuous net reclassification improvement (NRI) and integrated discrimination improvement (IDI) were calculated to quantify the incremental predictive value of preoperative ACAG beyond these conventional severity scores. Subsequently, Cox proportional hazards regression models were employed to evaluate the association between ACAG and 28-day mortality, analyzed both as a continuous variable and as a categorical variable. A total of six Cox regression models were constructed, each progressively incorporating more covariates: the unadjusted model included no covariates; Model 1 adjusted for baseline demographic characteristics; Model 2 further adjusted for comorbidities; Model 3 added treatment factors within 48 h of ICU admission; Model 4 incorporated vital signs and clinical scores within 48 h of ICU admission; Model 5 further included laboratory results within 48 h of ICU admission.To assess statistical model stability, we examined multicollinearity using the variance inflation factor (VIF), with a VIF > 10 indicating substantial collinearity. These diagnostics were used only to assess numerical stability of the extended models and were not considered evidence against possible over-adjustment [14]. Because postoperative severity scores and early postoperative laboratory variables may partly reflect downstream clinical states after surgery, the baseline-adjusted model was treated as the main inferential model, whereas models additionally incorporating postoperative severity scores and laboratory variables were interpreted as extended exploratory models used to assess the robustness and attenuation of the association. When the proportional hazards assumption was not met, the hazard ratio (HR) was interpreted as a time-weighted average over the entire follow-up period [15, 16].

To further clarify the association between ACAG and survival prognosis, we utilized restricted cubic spline (RCS) analysis with four knots in the six Cox models. Additionally, subgroup analyses were performed to investigate the relationship between ACAG and prognosis across various populations, with stratification based on variables such as age, body mass index, gender, chronic kidney disease, AKI, heart failure, myocardial infarction, ischemic heart disease, hypertension, stroke, chronic obstructive pulmonary disease, type 1 diabetes, type 2 diabetes, cancer, NSAID usage, antihypertensive medication usage, antibiotic usage, and corticosteroid usage. Finally, we performed exploratory mediation analyses to evaluate whether selected clinical variables might account for part of the observed association between preoperative ACAG and 28-day mortality. Specifically, the cumulative effect of ACAG was segmented into the average direct effect (ADE) on survival. Additionally, the average causal mediation effect (ACME) was mediated through intermediary variables. A mediation effect was considered significant when both the association between X and M, and M and Y were statistically significant [17]. Because this was a retrospective observational study, these analyses were considered hypothesis-generating, and the assumptions required for formal causal mediation analysis, particularly clear temporal ordering and the absence of unmeasured confounding, could not be fully verified. A two-tailed *p*-value of < 0.05 was considered statistically significant. All analyses in this study were conducted using R software (version 4.3.1).

## Results

### Study population

From the MIMIC-IV v3.0 database, 23,081 ICU patients who underwent cardiac surgery were initially considered. After applying exclusion criteria, including missing survival data, ACAG data, and abnormal baseline data, 5,006 patients were included in the final analysis.

### Baseline demographic and clinical characteristics

Patients were divided into three groups based on ACAG levels: low, normal, and high. Before analysis, variables with more than 20% missing data, including neutrophils, lymphocytes, albumin, total protein, C-reactive protein, temperature in Fahrenheit, and non-invasive blood pressure (NBPM), were excluded. Significant differences were observed across these groups. Older age and higher BMI were more common in the normal and high ACAG groups (*P* < 0.001 and *P* = 0.029, respectively). Clinical variables such as urea nitrogen, creatinine, and glucose were elevated in the high ACAG group, indicating poorer renal function and metabolic status (*P* < 0.001). SOFA and SAPSII scores were higher, reflecting more severe illness (*P* < 0.001). In the high ACAG group, other cardiac surgery was predominant (55.6%), followed by coronary artery surgery (25.8%), while valve surgery and arrhythmia device implantation accounted for 10.3% and 8.27%, respectively. This contrasted with the normal ACAG group, where coronary artery surgery was most common (42.0%). Higher mortality rates and shorter survival times were also significantly associated with the high ACAG group (*P* < 0.001), highlighting ACAG as a critical prognostic marker (Table 1).

**Table 1:** Baseline demographic and clinical characteristics of patients stratified by ACAG levels

	ACAG (< 12)	ACAG (12–20)	ACAG (> 20)	*P*
	*N* = 836	*N* = 3674	*N* = 496	
Age	68.0 [61.0;76.0]	70.0 [61.0;78.0]	70.0 [59.0;78.0]	<0.001
Body Mass Index	27.7 [24.4;31.4]	28.1 [24.8;32.2]	28.1 [24.4;32.0]	0.029
Insurance:				<0.001
Medicaid	65 (7.78%)	341 (9.28%)	63 (12.7%)	
Medicare	444 (53.1%)	2153 (58.6%)	304 (61.3%)	
Other	26 (3.11%)	86 (2.34%)	6 (1.21%)	
Private	301 (36.0%)	1094 (29.8%)	123 (24.8%)	
Race:				0.007
Asian	13 (1.56%)	64 (1.74%)	9 (1.81%)	
Black	27 (3.23%)	118 (3.21%)	21 (4.23%)	
Hispanic	17 (2.03%)	105 (2.86%)	14 (2.82%)	
Other	225 (26.9%)	1095 (29.8%)	183 (36.9%)	
White	554 (66.3%)	2292 (62.4%)	269 (54.2%)	
Marital status:				<0.001
Divorced	59 (7.06%)	301 (8.19%)	44 (8.87%)	
Married	585 (70.0%)	2230 (60.7%)	275 (55.4%)	
Single	139 (16.6%)	720 (19.6%)	106 (21.4%)	
Widowed	53 (6.34%)	423 (11.5%)	71 (14.3%)	
Gender:				<0.001
Female	197 (23.6%)	1133 (30.8%)	189 (38.1%)	
Male	639 (76.4%)	2541 (69.2%)	307 (61.9%)	
Urea nitrogen	14.3 [11.5;18.0]	17.0 [13.0;22.5]	27.0 [18.5;45.4]	<0.001
Creatinine	0.85 [0.70;1.00]	0.93 [0.75;1.20]	1.43 [0.95;2.35]	<0.001
Hemoglobin	10.6 [9.60;11.6]	10.5 [9.40;11.8]	10.4 [9.15;11.9]	0.214
Red blood cells	3.49 [3.12;3.84]	3.47 [3.12;3.90]	3.48 [3.04;4.01]	0.796
Platelet count	146 [123;178]	164 [132;207]	182 [131;248]	<0.001
Sodium	138 [136;140]	138 [136;140]	138 [135;140]	0.065
Chloride	107 [105;109]	106 [103;108]	103 [99.0;107]	<0.001
Glucose	118 [105;132]	122 [107;141]	144 [117;194]	<0.001
Potassium	4.30 [4.10;4.55]	4.30 [4.05;4.60]	4.30 [3.95;4.77]	0.911
White blood cells	11.9 [9.46;14.7]	12.3 [9.68;15.5]	13.3 [9.70;17.0]	<0.001
GCS score	15.0 [14.0;15.0]	15.0 [14.0;15.0]	15.0 [14.0;15.0]	0.694
SOFA score	4.00 [3.00;6.00]	5.00 [3.00;7.00]	7.00 [4.00;10.0]	<0.001
SAPSII score	32.0 [25.0;39.2]	35.0 [28.0;42.0]	42.0 [34.0;53.0]	<0.001
Heart rate	79.5 [73.5;84.7]	80.3 [73.8;88.1]	85.1 [74.9;95.3]	<0.001
Respiratory rate	17.4 [15.8;19.0]	17.9 [16.2;20.0]	19.5 [17.3;22.0]	<0.001
Spo2	97.6 [96.5;98.6]	97.6 [96.4;98.6]	97.0 [95.6;98.5]	<0.001
AKI:				<0.001
No	239 (28.6%)	944 (25.7%)	83 (16.7%)	
Yes	597 (71.4%)	2730 (74.3%)	413 (83.3%)	
CKD:				<0.001
No	743 (88.9%)	3009 (81.9%)	347 (70.0%)	
Yes	93 (11.1%)	665 (18.1%)	149 (30.0%)	
Heart failure:				<0.001
No	660 (78.9%)	2343 (63.8%)	188 (37.9%)	
Yes	176 (21.1%)	1331 (36.2%)	308 (62.1%)	
Myocardial infarction:				<0.001
No	647 (77.4%)	2734 (74.4%)	318 (64.1%)	
Yes	189 (22.6%)	940 (25.6%)	178 (35.9%)	
IHD:				0.047
No	221 (26.4%)	872 (23.7%)	139 (28.0%)	
Yes	615 (73.6%)	2802 (76.3%)	357 (72.0%)	
Hypertension:				<0.001
No	361 (43.2%)	1839 (50.1%)	348 (70.2%)	
Yes	475 (56.8%)	1835 (49.9%)	148 (29.8%)	
Stroke:				0.665
No	775 (92.7%)	3385 (92.1%)	453 (91.3%)	
Yes	61 (7.30%)	289 (7.87%)	43 (8.67%)	
COPD:				0.002
No	761 (91.0%)	3261 (88.8%)	420 (84.7%)	
Yes	75 (8.97%)	413 (11.2%)	76 (15.3%)	
T1DM:				<0.001
No	830 (99.3%)	3641 (99.1%)	478 (96.4%)	
Yes	6 (0.72%)	33 (0.90%)	18 (3.63%)	
T2DM:				<0.001
No	640 (76.6%)	2447 (66.6%)	292 (58.9%)	
Yes	196 (23.4%)	1227 (33.4%)	204 (41.1%)	
Cancer:				0.624
No	727 (87.0%)	3173 (86.4%)	422 (85.1%)	
Yes	109 (13.0%)	501 (13.6%)	74 (14.9%)	
Ventilation:				<0.001
No	68 (8.13%)	423 (11.5%)	79 (15.9%)	
Yes	768 (91.9%)	3251 (88.5%)	417 (84.1%)	
NSAID Use:				<0.001
No	322 (38.5%)	1949 (53.0%)	338 (68.1%)	
Yes	514 (61.5%)	1725 (47.0%)	158 (31.9%)	
Antihypertensive Use:				0.002
No	299 (35.8%)	1309 (35.6%)	217 (43.8%)	
Yes	537 (64.2%)	2365 (64.4%)	279 (56.2%)	
Antibiotic Use:				<0.001
No	233 (27.9%)	1616 (44.0%)	224 (45.2%)	
Yes	603 (72.1%)	2058 (56.0%)	272 (54.8%)	
Glucocorticoid Use:				<0.001
No	830 (99.3%)	3572 (97.2%)	462 (93.1%)	
Yes	6 (0.72%)	102 (2.78%)	34 (6.85%)	
Death:				<0.001
No	823 (98.4%)	3462 (94.2%)	357 (72.0%)	
Yes	13 (1.56%)	212 (5.77%)	139 (28.0%)	
Surgery Type:				<0.001
Arrhythmia Device	201 (5.47%)	32 (3.83%)	41 (8.27%)	
Coronary Artery Surgery	1542 (42.0%)	385 (46.1%)	128 (25.8%)	
Other Cardiac Surgery	1389 (37.8%)	240 (28.7%)	276 (55.6%)	
Valve Surgery	542 (14.8%)	179 (21.4%)	51 (10.3%)	
OS	28.0 [28.0;28.0]	28.0 [28.0;28.0]	28.0 [18.9;28.0]	<0.001
ACAG	11.0 [10.0;11.5]	15.2 [13.8;17.0]	22.5 [21.0;24.5]	<0.001

*ACAG *Albumin-Corrected Anion Gap, *BMI *Body Mass Index, *GCS *Glasgow Coma Scale, *SOFA *Sequential Organ Failure Assessment, *SAPSII *Simplified Acute Physiology Score II, *AKI *Acute Kidney Injury, *CKD *Chronic Kidney Disease, *IHD *Ischemic Heart Disease, *COPD *Chronic Obstructive Pulmonary Disease, *T1DM *Type 1 Diabetes Mellitus, *T2DM *Type 2 Diabetes Mellitus, *NSAID *Non-Steroidal Anti-Inflammatory Drug, *OS *Overall Survival

### Survival analysis and prognostic value of ACAG

**Fig. 2:**
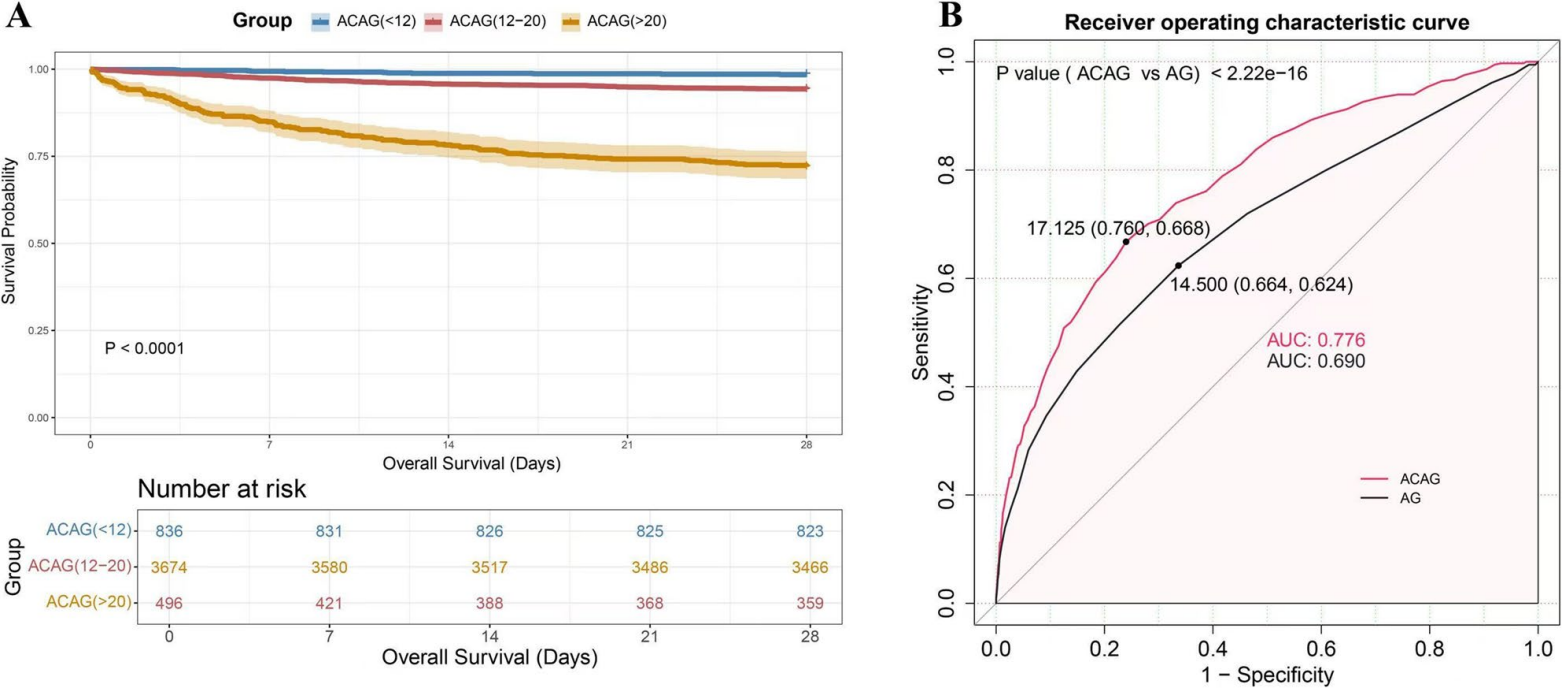
**A** Kaplan-Meier survival curves for 28-day overall survival across ACAG groups. Higher ACAG levels were associated with significantly lower survival rates (*P* < 0.0001). **B** ROC curves showing that ACAG is a better predictor of 28-day mortality compared to the anion gap, with a higher AUC (0.776 vs. 0.690, *P* < 2.22e-16)

The KM analysis revealed a notably reduced 28-day survival rate among individuals with elevated ACAG levels (> 20 mmol/l) compared to those with normal or lower levels (*P* < 0.0001) (Fig. 2). ROC analysis further indicated that ACAG offers greater predictive power for 28-day mortality (AUC = 0.776) compared to the conventional anion gap (AUC = 0.690). These findings suggest that higher ACAG levels are closely linked to worse short-term outcomes in ICU patients, emphasizing its clinical utility as a prognostic marker.

#### Incremental predictive value of preoperative ACAG beyond SOFA and SAPS II

To further evaluate whether preoperative ACAG provided prognostic information beyond established ICU severity scores, we compared baseline SOFA- and SAPS II-based models with corresponding models incorporating preoperative ACAG. As shown in Table 2, the addition of preoperative ACAG significantly improved model discrimination, with the AUC increasing from 0.673 to 0.796 for the SOFA-based model and from 0.755 to 0.823 for the SAPS II-based model (both DeLong test *P* < 0.001). Continuous net reclassification improvement (NRI) and integrated discrimination improvement (IDI) analyses also demonstrated significant incremental predictive value after adding preoperative ACAG (SOFA + ACAG: NRI = 0.677, IDI = 0.090, both *P* < 0.001; SAPS II + ACAG: NRI = 0.678, IDI = 0.092, both *P* < 0.001). These findings suggest that preoperative ACAG may provide additional risk-stratification information beyond established severity scores.

**Table 2:** Predictive performance of SOFA and SAPS II scores with and without preoperative ACAG

Prediction Models	AUC	*P*-value (DeLong)	Continuous NRI	*P*-value	IDI	*P*-value
SOFA Score	0.673					
SOFA Score+Preoperative ACAG	0.796	< 0.001	0.677	< 0.001	0.09	< 0.001
SAPS II Score	0.755					
SAPS II Score+ Preoperative ACAG	0.823	< 0.001	0.678	< 0.001	0.09	< 0.001

#### Cox regression results

Cox regression analysis indicated that elevated ACAG levels (> 20 mmol/l) were consistently linked to a markedly increased risk of mortality in all models. In the unadjusted model, high ACAG exhibited a robust association with mortality (HR 5.603, 95% CI 4.523–6.940, *P* < 0.001). This relationship remained significant, though attenuated, after adjusting for demographic factors, comorbidities, vital signs, and clinical scores in the fully adjusted Model 5 (HR 1.523, 95% CI 1.174–1.977, *P* = 0.002). The attenuation of the association after adjustment suggests that part of the prognostic information captured by preoperative ACAG overlaps with postoperative illness severity and related physiological disturbances. Conversely, lower ACAG levels (< 12 mmol/l) were linked to a reduced mortality risk, although this protective effect diminished with further adjustments (HR 0.520, 95% CI 0.294–0.919, *P* = 0.024) (Table 3).

**Table 3:** Cox regression results showing hazard ratios (HR) for mortality across different ACAG levels, with the reference group being ACAG 12–20 mmol/l

Model	ACAG		ACAG (12–20)	ACAG (< 12)		ACAG (> 20)	
	HR (95%CI)	*P* value		HR (95%CI)	*P* value	HR (95%CI)	*P* value
Unadjusted Model	1.225 (1.205–1.246)	< 0.001	Reference	0.264 (0.151–0.462)	< 0.001	5.603 (4.523–6.940)	< 0.001
Model 1	1.220 (1.199–1.242)	< 0.001	Reference	0.292 (0.166–0.511)	< 0.001	5.395 (4.348–6.693)	< 0.001
Model 2	1.193 (1.170–1.216)	< 0.001	Reference	0.332 (0.189–0.582)	< 0.001	4.090 (3.269–5.118)	< 0.001
Model 3	1.181 (1.158–1.204)	< 0.001	Reference	0.355 (0.202–0.624)	< 0.001	3.755 (2.997–4.705)	< 0.001
Model 4	1.094 (1.070–1.120)	< 0.001	Reference	0.473 (0.268–0.833)	0.010	1.947 (1.524–2.486)	< 0.001
Model 5	1.049 (1.024–1.075)	< 0.001	Reference	0.546 (0.309–0.964)	0.037	1.491 (1.151–1.931)	0.002

Unadjusted model: Includes no covariates

Model 1: Adjusts for baseline demographic characteristics, including age, body mass index, gender, race, marital status, and insurance type

Model 2: Further adjusts for comorbidities, including chronic kidney disease, acute kidney injury, heart failure, myocardial infarction, ischemic heart disease, hypertension, stroke, chronic obstructive pulmonary disease, type 1 diabetes mellitus, type 2 diabetes mellitus, and cancer

Model 3: Includes treatment factors within the first 48 h of ICU admission, including mechanical ventilation, NSAID use, antihypertensive use, antibiotic use, and glucocorticoid use

Model 4: Adds vital signs and clinical scores within the first 48 h, including heart rate, respiratory rate, oxygen saturation (SpO2), Glasgow Coma Scale (GCS), Sequential Organ Failure Assessment (SOFA), and Simplified Acute Physiology Score II (SAPSII)

Model 5: Further includes laboratory results within the first 48 h, such as blood urea nitrogen, creatinine, hemoglobin, red blood cell count, platelet count, sodium, chloride, glucose, potassium, white blood cell count and surgery type

### RCS analysis

**Fig. 3:**
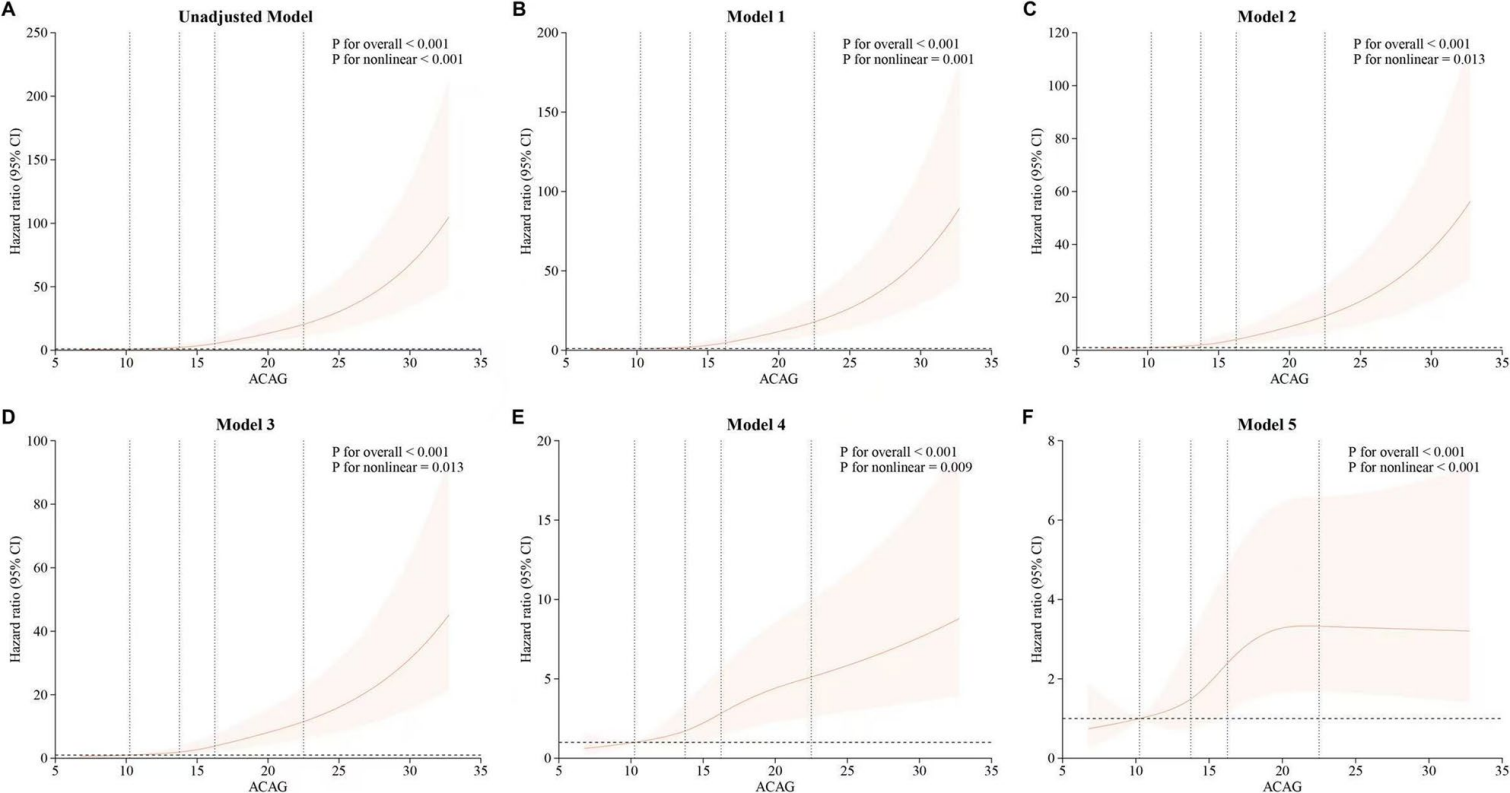
RCS analysis illustrating the nonlinear association between ACAG levels and mortality risk across different models. **A** Unadjusted model, (**B**) Model 1 (demographic adjustment), (**C**) Model 2 (added comorbidities), (**D**) Model 3 (treatment factors), (**E**) Model 4 (vital signs and clinical scores), and (**F**) Model 5 (fully adjusted). The analysis consistently shows an elevated mortality risk with ACAG levels above 20 mmol/l

**Fig. 4:**
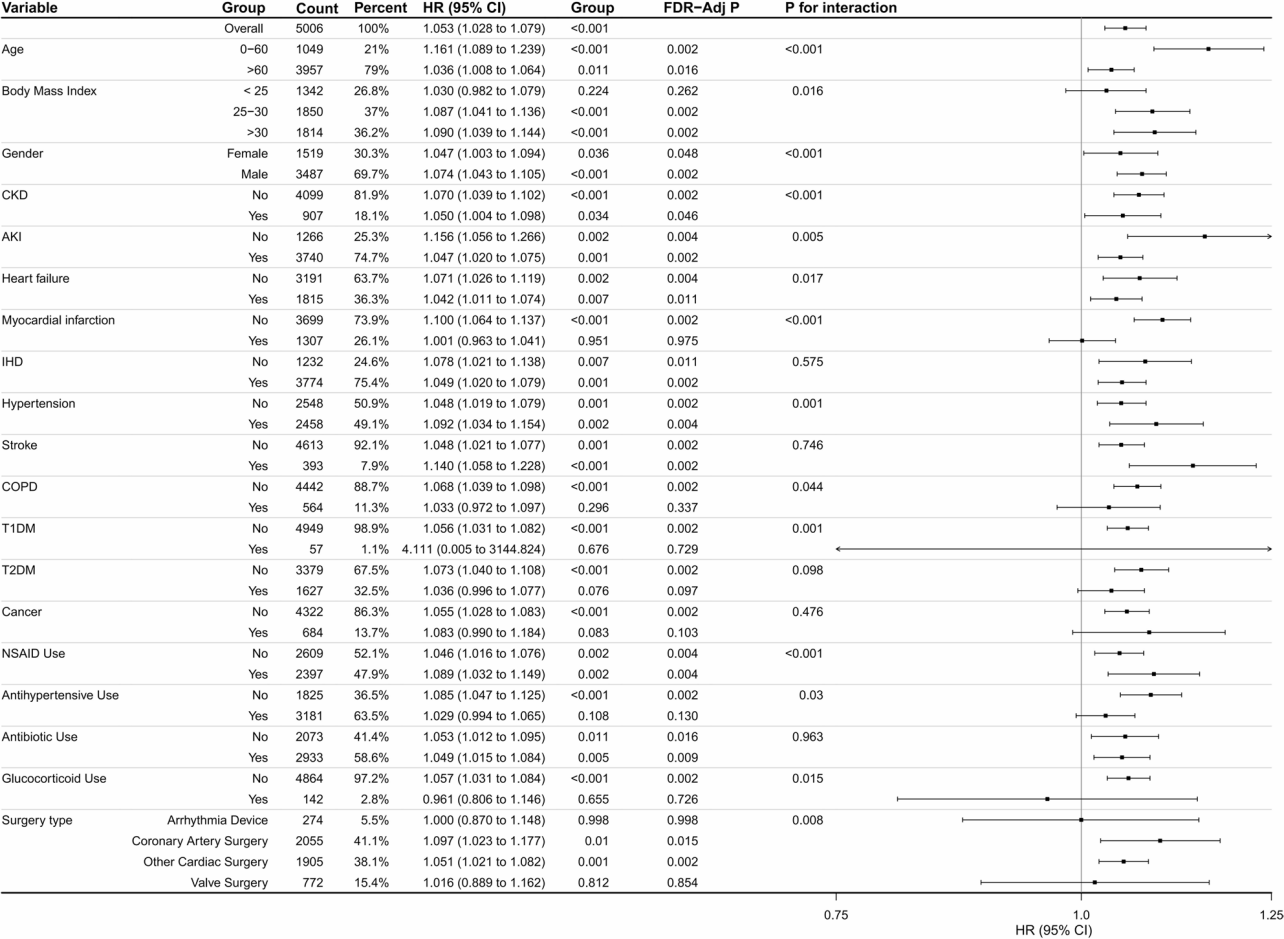
Exploratory subgroup analysis of the association between preoperative ACAG and 28-day mortality across clinically relevant strata. Hazard ratios and 95% confidence intervals are shown for each subgroup. These findings should be interpreted cautiously because of multiple comparisons

The RCS analysis demonstrated a clear non-linear association between ACAG levels and the risk of death (Fig. 3). Across all models, the risk of mortality significantly increased when ACAG levels surpassed 20 mmol/l. This association remained strong even after adjusting for comprehensive clinical and laboratory variables in Model 5 (Fig. 3). These results highlight the importance of monitoring ACAG levels in ICU patients, especially since levels exceeding 20 mmol/l are linked to a significantly increased risk of death (Fig. 4).

### Subgroup analysis

In exploratory subgroup analyses, the association between preoperative ACAG and 28-day mortality appeared to persist across multiple clinically relevant strata. In a stratified analysis according to CPB status, the association remained significant in both the CPB group (adjusted HR 1.223, 95% CI 1.169–1.281) and the non-CPB group (adjusted HR 1.148, 95% CI 1.123–1.174), and both findings remained significant after FDR correction (both FDR-adjusted *P* < 0.001; Supplementary Fig. S3). The corresponding Kaplan–Meier curves for the CPB and non-CPB subgroups are shown in Supplementary Figs. S1 and S2, respectively. These results suggest that the association was robust in this key surgical subgroup, although the overall subgroup findings should still be interpreted cautiously in light of multiple testing.

### Exploratory mediation analysis

**Fig. 5:**
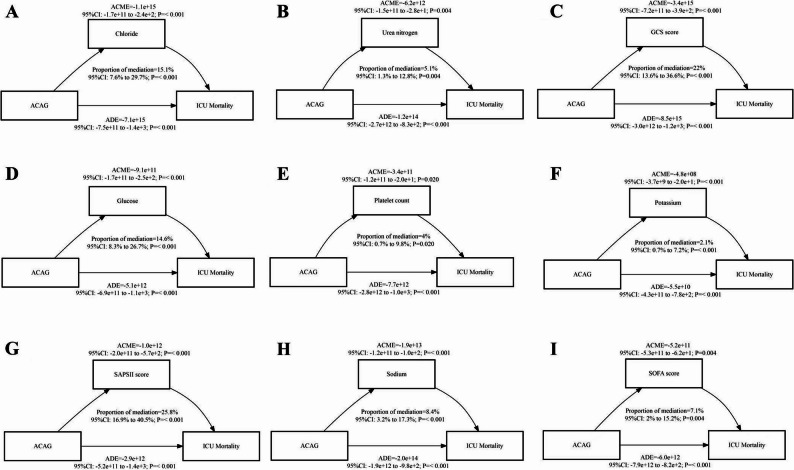
Exploratory mediation analysis of clinical variables that may account for part of the observed association between preoperative ACAG and 28-day mortality

Exploratory mediation analyses were performed to investigate potential clinical variables that might account for the observed association between preoperative ACAG and 28-day mortality following ICU admission. In these analyses, SAPS II and GCS scores accounted for the largest proportions of the association (25.8% and 22.0%, respectively). Other potential mediators, such as chloride (15.1%), glucose (14.6%), and urea nitrogen (5.1%), also accounted for significant, albeit smaller, proportions. These findings suggest potential interconnected pathophysiological pathways involving overall disease severity, neurological function, and electrolyte homeostasis, although definitive causal relationships cannot be inferred from this retrospective study(Fig. 5).

## Discussion

In this research, we explored the relationship between preoperative ACAG levels and 28-day mortality among patients who underwent cardiac surgery. Our findings indicate that higher ACAG levels, particularly those above 20 mmol/L, are significantly associated with poor prognosis in this patient population. This relationship persisted across multiple Cox regression models, whether ACAG was analyzed as a continuous or categorical variable. Notably, ACAG showed a stronger ability to predict 28-day mortality following ICU admission than the traditional AG. The areas under the ROC curve were 0.776 for ACAG and 0.690 for AG, respectively. The RCS analysis further clarified a non-linear association between ACAG and the risk of death, with a sharp rise in risk occurring at ACAG levels exceeding 20 mmol/L across all models.An additional point merits consideration regarding model interpretation. In our sequential Cox analyses, attenuation of the association after broader adjustment likely reflects partial overlap between preoperative ACAG and downstream illness-severity states after surgery. Because some postoperative severity scores and early postoperative laboratory variables may lie on the clinical pathway linking preoperative metabolic disturbance to short-term mortality, models incorporating these variables should be interpreted as conservative exploratory models rather than as the primary inferential model. We therefore consider the less extensively adjusted models, particularly those not including postoperative severity scores or postoperative laboratory variables, to be most informative for the main association of interest, while the more extensively adjusted models serve primarily to assess robustness and attenuation.

The enhanced prognostic value of ACAG over AG can be attributed to its correction for serum albumin levels. Hypoalbuminemia reduces the total protein concentration in the blood, which in turn affects the concentration and distribution of unmeasured anions [18]. By adjusting for albumin, ACAG provides a more comprehensive reflection of the metabolic status and electrolyte balance in cardiac surgery patients, thus offering greater sensitivity and reliability in predicting postoperative outcomes [19].Importantly, beyond merely outperforming the traditional anion gap, our continuous NRI and IDI analyses demonstrated that ACAG significantly improved the prognostic performance of established clinical models based on SOFA and SAPS II scores. This indicates that ACAG is not merely a surrogate of existing severity scores but provides additional and additive risk-stratification information. Similarly, a study in critically ill trauma patients demonstrated that higher ACAG levels within the first 24 h of admission were independently associated with increased 30-day and 90-day in-hospital mortality [20]. Another study emphasized the link between elevated ACAG and poor outcomes in cardiac arrest patients [21].These results suggest that ACAG may reflect common underlying metabolic disturbances or physiological stress responses that extend beyond specific disease states, potentially serving as a broadly applicable biomarker for risk stratification in acute care settings.The choice of 28-day mortality following ICU admission as the primary endpoint also deserves comment. In cardiac surgery patients, total hospital length of stay may vary substantially according to procedure type, postoperative recovery, rehabilitation needs, discharge planning, and non-fatal complications. Therefore, overall in-hospital mortality may be influenced not only by disease severity but also by hospitalization-related factors. By contrast, 28-day mortality following ICU admission focuses on the early high-acuity postoperative period, which is the time window most likely to be mechanistically linked to preoperative metabolic abnormalities such as elevated ACAG. We therefore considered this endpoint to be more appropriate for evaluating the short-term prognostic relevance of preoperative ACAG in critically ill cardiac surgery patients.

An elevated ACAG may increase the risk of mortality during ICU stays in cardiac surgery patients through several interrelated pathophysiological mechanisms. Fundamentally, an elevated ACAG indicates the accumulation of unmeasured anions, which is often associated with underlying metabolic acidosis and electrolyte imbalances [22]. Specifically, increased levels of ketone bodies and organic acids may contribute to a higher ACAG, suggesting underlying microcirculatory dysfunction that leads to the accumulation of metabolites and lactate [8, 23–25]. The resulting acidosis may amplify or prolong inflammatory responses by inhibiting neutrophil apoptosis mechanisms [26]. Moreover, an elevated ACAG may reflect impaired renal capacity to effectively regulate acid-base homeostasis, potentially complicating postoperative fluid management and metabolic balance [27]. These interconnected pathophysiological processes—metabolic acidosis, microcirculatory dysfunction, dysregulated inflammation, and renal impairment—may act synergistically to ultimately result in adverse outcomes during ICU stays in cardiac surgery patients.

In additional stratified analyses according to cardiopulmonary bypass (CPB) status, the prognostic association of preoperative ACAG remained significant in both the CPB and non-CPB subgroups even after false discovery rate (FDR) correction (Supplementary Figs. S1—S3). This finding is consistent with and extends previous work by Wang et al., who reported the prognostic value of preoperative ACAG in patients undergoing coronary artery bypass grafting (CABG). However, because the non-CPB subgroup still included procedurally heterogeneous patients, these stratified findings should be interpreted as supportive but exploratory. Regarding other clinical strata (such as age, gender, and baseline comorbidities), exploratory Subgroup analysis indicated that elevated ACAG levels showed a trend of being associated with a higher risk of death in most clinical subgroups, with particularly pronounced effects observed in younger patients (≤ 60 years), individuals with higher BMI (≥ 25), males, and patients without specific comorbidities such as AKI, CKD, HF, MI, or COPD. However, because these findings were derived from multiple exploratory comparisons without FDR correction, they must be interpreted cautiously as hypothesis-generating. If these differential associations are validated in future studies, several speculative mechanisms might explain them. A prospective study similarly found that younger patients had poorer outcomes after undergoing cardiac surgery [28]. The age-related differences might stem from the fact that younger patients requiring cardiac surgery often present with more severe or complex cases, potentially involving underlying genetic or metabolic disorders. The observed lower risk in females compared to males could theoretically be related to the cardioprotective effects of estrogen [29]. Regarding comorbidities, the higher mortality risk in patients without AKI compared to those with AKI could be explained by the more rigorous acid-base balance monitoring and management that AKI patients typically receive [30]. Similarly, the differential effects in patients without CKD, HF, MI, or COPD may reflect varying levels of medical attention and intervention strategies. Among different types of cardiac surgery, the strongest association was observed in patients undergoing coronary artery surgery. This may be due to elevated ACAG reflecting underlying metabolic acidosis, which could exacerbate myocardial ischemia during coronary procedures and impair tissue oxygen delivery [31]. Notably, patients who did not receive postoperative antibiotic treatment showed a higher risk of adverse outcomes. These results suggest that timely postoperative interventions, including antibiotic administration and blood pressure control, may play a role in patient management [32, 33]. Nevertheless, the possibility of residual multiplicity and type I error in these non-prespecified subgroups cannot be excluded, requiring further validation in independent cohorts. 

Exploratory mediation analysis suggested potential pathways associated with the relationship between ACAG and mortality in ICU patients. Key potential mediators included SAPSII scores (25.8%), GCS scores (22%), chloride (15.1%), glucose (14.6%), sodium (8.4%), SOFA scores (7.1%), urea nitrogen (5.1%), platelet count (4%), and potassium (2.1%). The prominent proportions accounted for by GCS and SAPSII scores as mediators suggest that the electrolyte imbalances represented by elevated ACAG levels may increase mortality risk by affecting patients’ neurological function and overall disease severity [34]. The mediation effects of electrolytes (chloride, sodium, and potassium) indicate that high ACAG levels might influence outcomes through their impact on acid-base balance and electrolyte homeostasis. The mediating roles of glucose and urea nitrogen imply potential links between ACAG and disturbances in glucose metabolism and liver function [35]. The involvement of platelet count as a mediator may reflect alterations in coagulation function [36].

These findings have significant clinical implications. ACAG shows considerable potential as a preoperative risk stratification tool for cardiac surgery patients. Incorporating ACAG into existing risk assessment models could enhance predictive accuracy. For patients with elevated ACAG, more comprehensive preoperative optimization should be considered, such as improving electrolyte balance and glucose control. Close monitoring of ACAG changes during the perioperative period could guide fluid management and metabolic balance. ACAG-guided interventions, such as early renal replacement therapy or more aggressive hemodynamic management, may improve patient outcomes, although further research is needed to validate these approaches.

The strengths of our study include the extensive patient cohort derived from the MIMIC-IV, enhancing the reliability and generalizability of the results. We conducted comprehensive adjustments for potential confounding factors, increasing the credibility of our findings. The innovative application of restricted cubic spline analysis and mediation analysis provided in-depth exploration of the nonlinear relationship between ACAG and mortality risk, as well as its mechanisms of action, offering new perspectives on the prognostic value of ACAG in cardiac surgery patients.

However, several limitations should be acknowledged. The retrospective design of this study may introduce residual confounding. The MIMIC-IV database, being from a single center, may limit the generalizability of the results. Potential coding errors in the database could affect the accuracy of the findings. We excluded variables with more than 20% missing data, which may have led to the omission of some important factors, impacting the comprehensiveness of the study results. Furthermore, the mediation analyses in this study should be interpreted with caution. Because of the retrospective observational design, the assumptions required for causal mediation analysis, particularly clear temporal ordering and the absence of unmeasured confounding between the exposure, mediator, and outcome, cannot be fully verified, meaning these findings cannot serve as confirmatory evidence of causal pathways. Finally, the subgroup analyses involved multiple comparisons, which may increase the risk of type I error. Although FDR correction supported the robustness of the CPB/non-CPB stratified findings, the overall subgroup results should still be regarded as exploratory and hypothesis-generating.

Future research directions should include prospective studies to further validate the prognostic value of ACAG in cardiac surgery patients. Investigations into potential interventions, such as aggressive acid-base balance management, to modify ACAG levels and assess their impact on patient outcomes are warranted. In-depth studies on the potential mechanisms linking ACAG with postoperative complications, such as inflammatory responses and organ dysfunction, would contribute to a better understanding of the clinical significance of ACAG.

## Conclusion

In conclusion, elevated preoperative ACAG levels were significantly associated with increased 28-day mortality risk in cardiac surgery patients. As a simple, readily available, and cost-effective indicator, ACAG may serve as a useful early risk-stratification marker and could complement existing prognostic assessment frameworks. However, because part of this association may reflect overall illness severity and postoperative physiological stress, its causal role cannot be inferred from this observational study. Further prospective studies are needed to validate these findings and to determine whether incorporating ACAG into perioperative risk assessment can improve clinical decision-making.

## Supplementary Information

Supplementary Material 1.

Supplementary Material 2.

## Data Availability

The data that support the findings of this study are available in the MIMIC-IV database at https://physionet.org/content/mimiciv/3.0/. Access to the database requires registration and completion of the required training (CITI program).

## Abbreviations

ACAG: Albumin-corrected anion gap
AG: Anion gap
MIMIC-IV: Medical Information Mart for Intensive Care IV
ICU: Intensive care unit
BIDMC: Beth Israel Deaconess Medical Center
IRB: Institutional Review Board
ROC: Receiver operating characteristic
AUC: Area under the curve
RCS: Restricted cubic spline
HR: Hazard ratio
CI: Confidence interval
GCS: Glasgow Coma Scale
SAPS II: Simplified Acute Physiology Score II
SOFA: Sequential Organ Failure Assessment
AKI: Acute kidney injury
CKD: Chronic kidney disease
