# Supplementary material for: Association of preoperative albumin-corrected anion gap with 28-day mortality in cardiac surgery patients: a retrospective cohort study
Source: BMC Cardiovasc Disord. 2026 Mar 27;26:397. doi: 10.1186/s12872-026-05779-9 (PMC13147607; doi:10.1186/s12872-026-05779-9)
Supplement: Supplementary file 1 — Supplementary Material 1. [file 12872_2026_5779_MOESM1_ESM.docx]

**
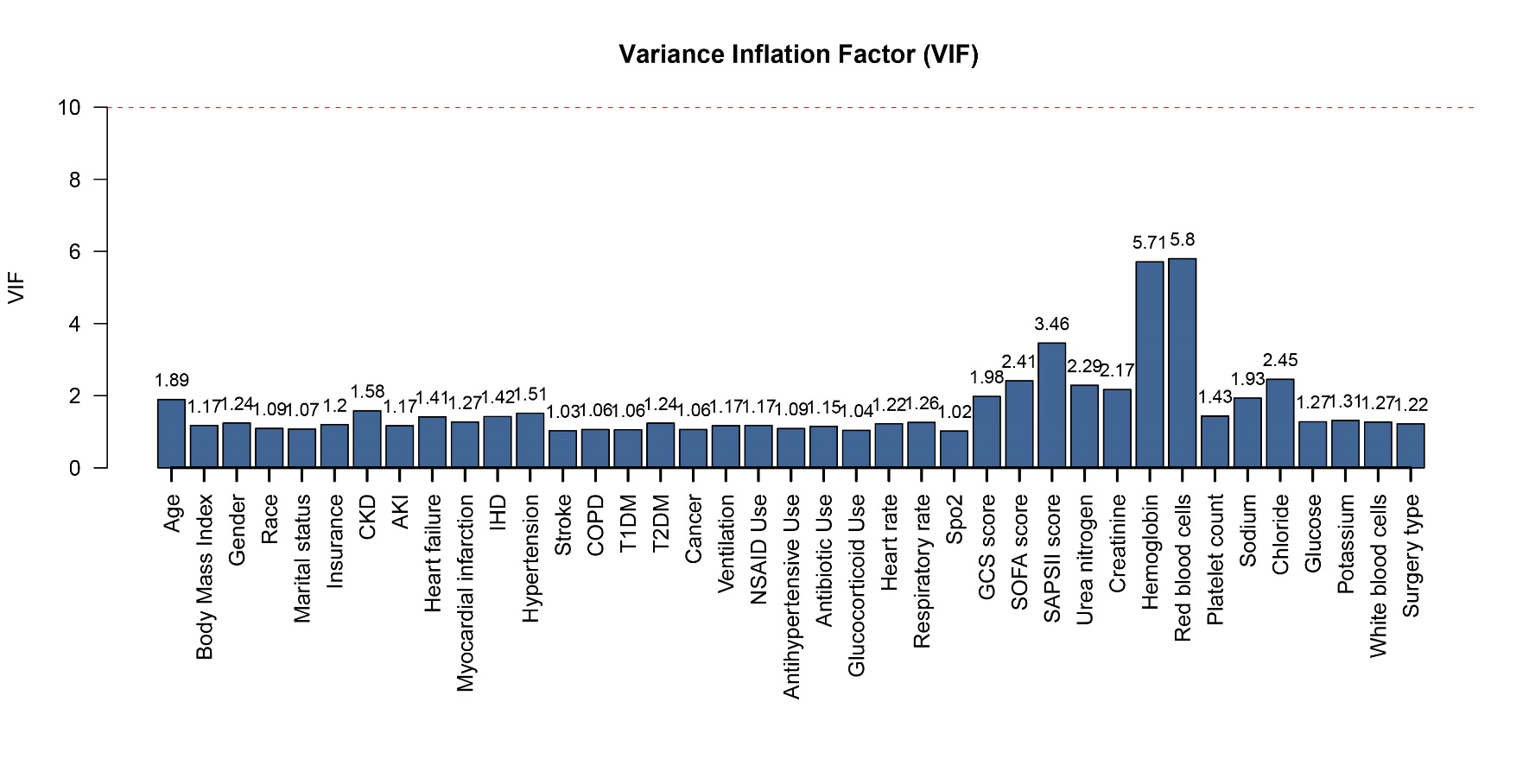
**

**Supplementary Figure 1.** Variance Inflation Factor (VIF) analysis for detecting multicollinearity among the variables included in the Cox regression model. The VIF values for each variable are displayed, with a value greater than 10 indicating potential multicollinearity.

**Table S1.** Procedures and Corresponding ICD Codes in Cardiac Surgery

| **ICD description** | **ICD code** | **ICD version** |
| --- | --- | --- |
| **Percutaneous transluminal coronary angioplasty [PTCA]** | 66 | 9 |
| **Extracorporeal circulation auxiliary to open heart surgery** | 3961 | 9 |
| **Insertion of drug-eluting coronary artery stent(s)** | 3607 | 9 |
| **Single internal mammary-coronary artery bypass** | 3615 | 9 |
| **Bypass coronary artery, one artery from left internal mammary, open approach** | 02100Z9 | 10 |
| **Excision or destruction of other lesions or tissue of the heart, endovascular approach** | 3734 | 9 |
| **Open and other replacement of aortic valve with tissue graft** | 3521 | 9 |
| **Insertion of non-drug-eluting coronary artery stent(s)** | 3606 | 9 |
| **Aortocoronary bypass of two coronary arteries** | 3612 | 9 |
| **Aortocoronary bypass of three coronary arteries** | 3613 | 9 |
| **Dilation of the coronary artery, one artery with a drug-eluting intraluminal device, percutaneous approach** | 027034Z | 10 |
| **Initial insertion of dual-chamber device** | 3783 | 9 |
| **Initial insertion of transvenous leads [electrodes] into the atrium and ventricle** | 3772 | 9 |
| **Replacement of aortic valve with zooplastic tissue, open approach** | 02RF08Z | 10 |
| **Bypass coronary artery, two arteries from the aorta with autologous venous tissue, open approach** | 021109W | 10 |
| **Aortocoronary bypass of one coronary artery** | 3611 | 9 |
| **Insertion of pacemaker lead into the right atrium, percutaneous approach** | 02H63JZ | 10 |
| **Insertion of pacemaker, dual-chamber into subcutaneous tissue and fascia of the chest, open approach** | 0JH606Z | 10 |
| **Replacement of aortic valve with zooplastic tissue, percutaneous approach** | 02RF38Z | 10 |
| **Bypass coronary artery, three arteries from the aorta with autologous venous tissue, open approach** | 021209W | 10 |
| **Implantation or replacement of automatic cardioverter/defibrillator, total system [AICD]** | 3794 | 9 |
| **Bypass coronary artery, one artery from the aorta with autologous venous tissue, open approach** | 021009W | 10 |
| **Implant of pulsation balloon** | 3761 | 9 |
| **Open and other replacement of aortic valve** | 3522 | 9 |
| **Pericardiocentesis** | 370 | 9 |
| **Open heart valvuloplasty of mitral valve without replacement** | 3512 | 9 |
| **Aortocoronary bypass of four or more coronary arteries** | 3614 | 9 |
| **Supplement mitral valve with synthetic substitute, open approach** | 02UG0JZ | 10 |
| **Procedure on vessel bifurcation** | 44 | 9 |
| **Insertion of one vascular stent** | 45 | 9 |
| **Insertion of two vascular stents** | 46 | 9 |
| **Insertion of three vascular stents** | 47 | 9 |
| **Assistance with cardiac output using a balloon pump, continuous** | 5A02210 | 10 |
| **Automatic implantable cardioverter/defibrillator (AICD) check** | 8949 | 9 |
| **Bypass coronary artery, one artery from the aorta with autologous arterial tissue, open approach** | 02100AW | 10 |
| **Intraoperative cardiac pacemaker** | 3964 | 9 |
| **Drainage of the pericardial cavity with drainage device, percutaneous approach** | 0W9D30Z | 10 |
| **Fluoroscopy of multiple coronary artery bypass grafts using other contrast** | B213YZZ | 10 |
| **Insertion of a temporary transvenous pacemaker system** | 3778 | 9 |
| **Dilation of coronary artery, one artery, percutaneous approach** | 02703ZZ | 10 |
| **Insertion of four or more vascular stents** | 48 | 9 |
| **Endovascular replacement of aortic valve** | 3505 | 9 |
| **Bypass coronary artery, one artery from right internal mammary, open approach** | 02100Z8 | 10 |
| **Dilation of the coronary artery, one artery with two drug-eluting intraluminal devices, percutaneous approach** | 027035Z | 10 |
| **Implantation of cardiac resynchronization defibrillator, total system [CRT-D]** | 51 | 9 |
| **Insertion of a defibrillator lead into the right ventricle, percutaneous approach** | 02HK3KZ | 10 |
| **Replacement of aortic valve with synthetic substitute, open approach** | 02RF0JZ | 10 |
| **Percutaneous balloon valvuloplasty** | 3596 | 9 |
| **Excision, destruction, or exclusion of left atrial appendage (LAA)** | 3736 | 9 |
| **Insertion of defibrillator generator into chest subcutaneous tissue and fascia, open approach** | 0JH608Z | 10 |
| **Open and other replacement of mitral valve with tissue graft** | 3523 | 9 |
| **Excision or destruction of other lesions or tissue of the heart, open approach** | 3733 | 9 |
| **Removal of cardiac rhythm-related device from trunk subcutaneous tissue and fascia, open approach** | 0JPT0PZ | 10 |
| **Resection of vessel with replacement, aorta, abdominal** | 3844 | 9 |
| **Excision of mitral valve, open approach** | 02BG0ZZ | 10 |
| **Initial insertion of transvenous lead [electrode] into the ventricle** | 3771 | 9 |
| **Replacement of mitral valve with zooplastic tissue, open approach** | 02RG08Z | 10 |
| **Replacement of thoracic aorta, ascending/arch with synthetic substitute, open approach** | 02RX0JZ | 10 |
| **Assistance with cardiac output using an impeller pump, continuous** | 5A0221D | 10 |
| **Bypass coronary artery, two arteries from left internal mammary, open approach** | 02110Z9 | 10 |
| **Pericardiotomy** | 3712 | 9 |
| **Removal of cardiac lead from heart, percutaneous approach** | 02PA3MZ | 10 |
| **Supplement tricuspid valve with synthetic substitute, open approach** | 02UJ0JZ | 10 |
| **Extirpation of matter from coronary artery, one artery, percutaneous approach** | 02C03ZZ | 10 |
| **Bypass coronary artery, four or more arteries from the aorta with autologous venous tissue, open approach** | 021309W | 10 |
| **Revision or relocation of cardiac device pocket** | 3779 | 9 |
| **Implantation of cardiac resynchronization pacemaker without mention of defibrillation, total system [CRT-P]** | 50 | 9 |
| **Replacement of any type of pacemaker device with a dual-chamber device** | 3787 | 9 |
| **Supplement mitral valve with synthetic substitute, percutaneous approach** | 02UG3JZ | 10 |
| **Initial insertion of single-chamber device, not specified as rate responsive** | 3781 | 9 |
| **Insertion of short-term external heart assist system into heart, percutaneous approach** | 02HA3RZ | 10 |
| **Occlusion of left atrial appendage with extraluminal device, open approach** | 02L70CK | 10 |
| **Fluoroscopy of single coronary artery bypass graft using other contrast** | B212YZZ | 10 |

| **Table S2.** Proportional Hazards (PH) Assumption Testing Results | | | | |
| --- | --- | --- | --- | --- |
| **Model** | chisq | df | P | Variable |
| **Udjusted Model** | 3.805557 | 2 | 0.149 | GLOBAL |
|  |  |  |  |  |
| **Model 1** | 0.802081 | 1 | 0.37 | Age |
| **Model 1** | 6.78562 | 1 | 0.009 | Body_Mass_Index |
| **Model 1** | 0.067869 | 1 | 0.794 | Gender |
| **Model 1** | 6.213647 | 4 | 0.184 | Race |
| **Model 1** | 1.631074 | 3 | 0.652 | Marital_status |
| **Model 1** | 0.563962 | 3 | 0.905 | Insurance |
| **Model 1** | 18.89327 | 15 | 0.219 | GLOBAL |
|  |  |  |  |  |
| **Model 2** | 1.185608 | 1 | 0.276 | Age |
| **Model 2** | 6.758155 | 1 | 0.009 | Body_Mass_Index |
| **Model 2** | 0.064738 | 1 | 0.799 | Gender |
| **Model 2** | 6.14771 | 4 | 0.188 | Race |
| **Model 2** | 1.796405 | 3 | 0.616 | Marital_status |
| **Model 2** | 0.595991 | 3 | 0.897 | Insurance |
| **Model 2** | 0.113967 | 1 | 0.736 | CKD |
| **Model 2** | 1.174011 | 1 | 0.279 | AKI |
| **Model 2** | 4.23161 | 1 | 0.04 | Heart_failure |
| **Model 2** | 0.535585 | 1 | 0.464 | Myocardial_infarction |
| **Model 2** | 3.11186 | 1 | 0.078 | IHD |
| **Model 2** | 0.479548 | 1 | 0.489 | Hypertension |
| **Model 2** | 1.007104 | 1 | 0.316 | Stroke |
| **Model 2** | 1.434877 | 1 | 0.231 | COPD |
| **Model 2** | 0.882289 | 1 | 0.348 | T1DM |
| **Model 2** | 0.143006 | 1 | 0.705 | T2DM |
| **Model 2** | 0.978535 | 1 | 0.323 | Cancer |
| **Model 2** | 35.34866 | 26 | 0.104 | GLOBAL |
|  |  |  |  |  |
| **Model 3** | 1.221225 | 1 | 0.269 | Age |
| **Model 3** | 7.113388 | 1 | 0.008 | Body_Mass_Index |
| **Model 3** | 0.060073 | 1 | 0.806 | Gender |
| **Model 3** | 6.166197 | 4 | 0.187 | Race |
| **Model 3** | 1.882102 | 3 | 0.597 | Marital_status |
| **Model 3** | 0.589847 | 3 | 0.899 | Insurance |
| **Model 3** | 0.125041 | 1 | 0.724 | CKD |
| **Model 3** | 1.081126 | 1 | 0.298 | AKI |
| **Model 3** | 4.259174 | 1 | 0.039 | Heart_failure |
| **Model 3** | 0.558651 | 1 | 0.455 | Myocardial_infarction |
| **Model 3** | 3.100815 | 1 | 0.078 | IHD |
| **Model 3** | 0.49836 | 1 | 0.48 | Hypertension |
| **Model 3** | 1.096189 | 1 | 0.295 | Stroke |
| **Model 3** | 1.557874 | 1 | 0.212 | COPD |
| **Model 3** | 0.871534 | 1 | 0.351 | T1DM |
| **Model 3** | 0.165889 | 1 | 0.684 | T2DM |
| **Model 3** | 1.039401 | 1 | 0.308 | Cancer |
| **Model 3** | 3.093328 | 1 | 0.079 | Ventilation |
| **Model 3** | 11.70898 | 1 | <0.001 | NSAID_Use |
| **Model 3** | 0.010024 | 1 | 0.92 | Antihypertensive_Use |
| **Model 3** | 5.24347 | 1 | 0.022 | Antibiotic_Use |
| **Model 3** | 0.459813 | 1 | 0.498 | Glucocorticoid_Use |
| **Model 3** | 54.97104 | 31 | 0.005 | GLOBAL |
|  |  |  |  |  |
| **Model 4** | 0.887348 | 1 | 0.346 | Age |
| **Model 4** | 5.460289 | 1 | 0.019 | Body_Mass_Index |
| **Model 4** | 0.235467 | 1 | 0.627 | Gender |
| **Model 4** | 6.369803 | 4 | 0.173 | Race |
| **Model 4** | 2.056365 | 3 | 0.561 | Marital_status |
| **Model 4** | 1.522264 | 3 | 0.677 | Insurance |
| **Model 4** | 0.005909 | 1 | 0.939 | CKD |
| **Model 4** | 0.442483 | 1 | 0.506 | AKI |
| **Model 4** | 7.475138 | 1 | 0.006 | Heart_failure |
| **Model 4** | 0.006293 | 1 | 0.937 | Myocardial_infarction |
| **Model 4** | 2.232781 | 1 | 0.135 | IHD |
| **Model 4** | 0.122544 | 1 | 0.726 | Hypertension |
| **Model 4** | 1.341051 | 1 | 0.247 | Stroke |
| **Model 4** | 2.427806 | 1 | 0.119 | COPD |
| **Model 4** | 1.367274 | 1 | 0.242 | T1DM |
| **Model 4** | 0.259709 | 1 | 0.61 | T2DM |
| **Model 4** | 1.096711 | 1 | 0.295 | Cancer |
| **Model 4** | 3.451487 | 1 | 0.063 | Ventilation |
| **Model 4** | 8.489449 | 1 | 0.004 | NSAID_Use |
| **Model 4** | 0.010283 | 1 | 0.919 | Antihypertensive_Use |
| **Model 4** | 2.226414 | 1 | 0.136 | Antibiotic_Use |
| **Model 4** | 2.61328 | 1 | 0.106 | Glucocorticoid_Use |
| **Model 4** | 2.448079 | 1 | 0.118 | Heart_rate |
| **Model 4** | 1.452833 | 1 | 0.228 | Respiratory_rate |
| **Model 4** | 4.007666 | 1 | 0.045 | Spo2 |
| **Model 4** | 2.043191 | 1 | 0.153 | GCS_score |
| **Model 4** | 24.53733 | 1 | <0.001 | SOFA_score |
| **Model 4** | 14.82016 | 1 | <0.001 | SAPSII_score |
| **Model 4** | 90.34501 | 37 | <0.001 | GLOBAL |
|  |  |  |  |  |
| **Model 5** | 1.778184 | 1 | 0.252 | Age |
| **Model 5** | 5.314562 | 1 | 0.029 | Body_Mass_Index |
| **Model 5** | 0.067481 | 1 | 0.758 | Gender |
| **Model 5** | 6.561343 | 4 | 0.177 | Race |
| **Model 5** | 2.297896 | 3 | 0.543 | Marital_status |
| **Model 5** | 1.224311 | 3 | 0.784 | Insurance |
| **Model 5** | 0.056303 | 1 | 0.908 | CKD |
| **Model 5** | 0.426048 | 1 | 0.451 | AKI |
| **Model 5** | 7.842157 | 1 | 0.005 | Heart_failure |
| **Model 5** | 0.000782 | 1 | 0.766 | Myocardial_infarction |
| **Model 5** | 1.490118 | 1 | 0.281 | IHD |
| **Model 5** | 0.127984 | 1 | 0.728 | Hypertension |
| **Model 5** | 1.200564 | 1 | 0.323 | Stroke |
| **Model 5** | 1.789077 | 1 | 0.215 | COPD |
| **Model 5** | 1.530481 | 1 | 0.215 | T1DM |
| **Model 5** | 0.131617 | 1 | 0.688 | T2DM |
| **Model 5** | 1.416519 | 1 | 0.211 | Cancer |
| **Model 5** | 3.838354 | 1 | 0.073 | Ventilation |
| **Model 5** | 8.38631 | 1 | 0.01 | NSAID_Use |
| **Model 5** | 0.001679 | 1 | 0.961 | Antihypertensive_Use |
| **Model 5** | 2.412261 | 1 | 0.18 | Antibiotic_Use |
| **Model 5** | 3.789692 | 1 | 0.124 | Glucocorticoid_Use |
| **Model 5** | 1.552598 | 1 | 0.338 | Heart_rate |
| **Model 5** | 1.419659 | 1 | 0.151 | Respiratory_rate |
| **Model 5** | 0.7391 | 1 | 0.471 | Spo2 |
| **Model 5** | 2.296253 | 1 | 0.225 | GCS_score |
| **Model 5** | 21.42886 | 1 | <0.001 | SOFA_score |
| **Model 5** | 13.25339 | 1 | <0.001 | SAPSII_score |
| **Model 5** | 0.111718 | 1 | 0.934 | Urea_nitrogen |
| **Model 5** | 0.905636 | 1 | 0.273 | Creatinine |
| **Model 5** | 0.182648 | 1 | 0.71 | Hemoglobin |
| **Model 5** | 0.092947 | 1 | 0.829 | Red_blood_cells |
| **Model 5** | 3.097942 | 1 | 0.072 | Platelet_count |
| **Model 5** | 0.139172 | 1 | 0.87 | Sodium |
| **Model 5** | 0.297378 | 1 | 0.74 | Chloride |
| **Model 5** | 3.3052 | 1 | 0.066 | Glucose |
| **Model 5** | 2.72812 | 1 | 0.088 | Potassium |
| **Model 5** | 6.202189 | 1 | 0.022 | White_blood_cells |
| **Model 5** | 0.81919 | 3 | 0.845 | Surgery_type |
| **Model 5** | 89.075 | 47 | 0.001 | GLOBAL |
