# Supplementary material for: Association of preoperative albumin-corrected anion gap with 28-day mortality in cardiac surgery patients: a retrospective cohort study
Source: BMC Cardiovasc Disord. 2026 Mar 27;26:397. doi: 10.1186/s12872-026-05779-9 (PMC13147607; doi:10.1186/s12872-026-05779-9)
Supplement: Supplementary file 2 — Supplementary Material 2. [file 12872_2026_5779_MOESM2_ESM.docx]

Supplementary Figure S1


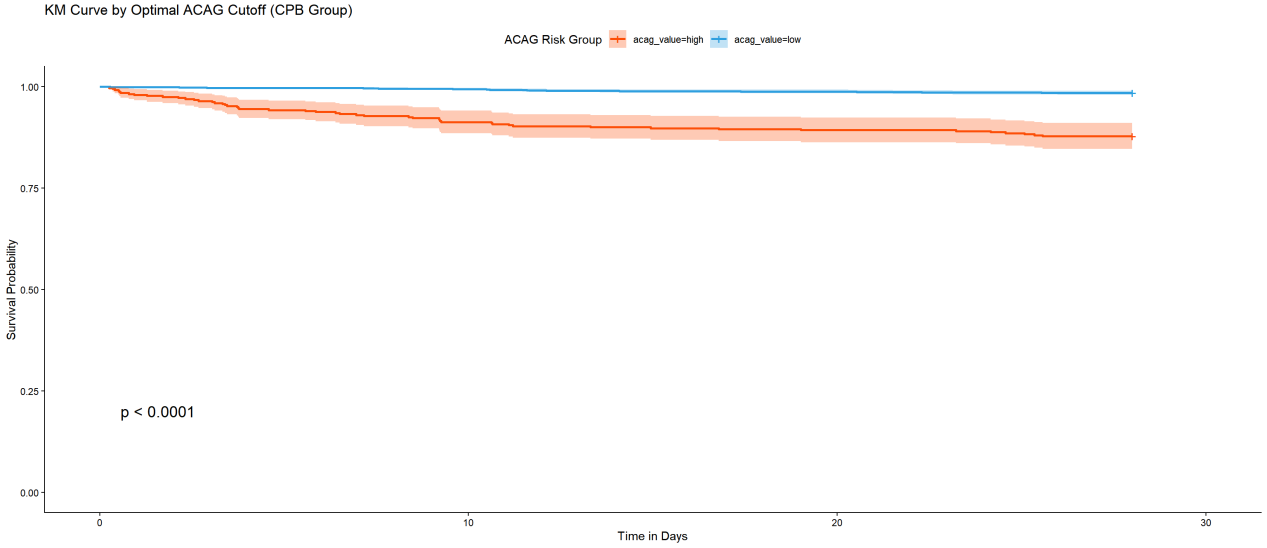


**Supplementary Figure S1.** Kaplan–Meier curve for 28-day mortality according to preoperative ACAG category in the CPB subgroup.

Supplementary Figure S2


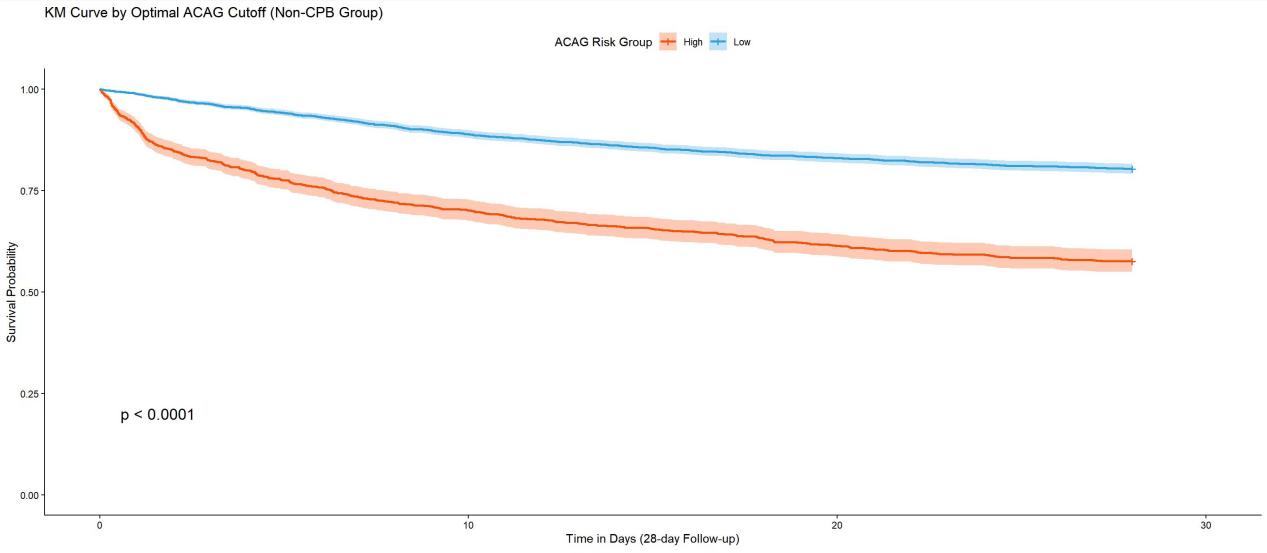


****Supplementary Figure S2.**** Kaplan–Meier curve for 28-day mortality according to preoperative ACAG category in the non-CPB subgroup

Supplementary Figure S3


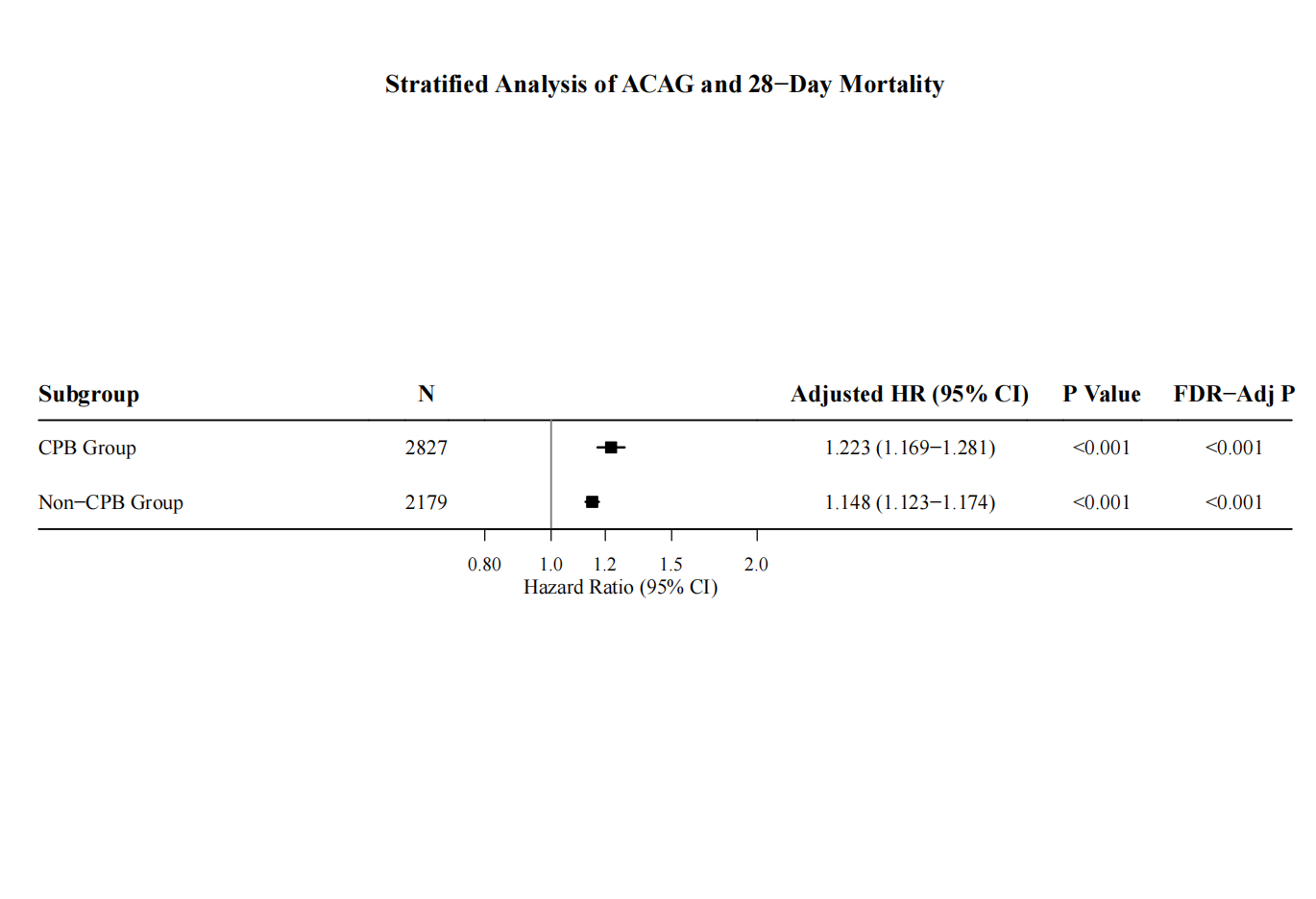


**Supplementary Figure S3.** Exploratory stratified analysis of the association between preoperative ACAG and 28-day mortality according to cardiopulmonary bypass (CPB) status. Adjusted hazard ratios (HRs), 95% confidence intervals (CIs), and false discovery rate (FDR)-adjusted P values are shown for the CPB and non-CPB groups.
